# Adult Age Differences in Effects of Text Spacing on Eye Movements During Reading

**DOI:** 10.3389/fpsyg.2018.02700

**Published:** 2019-01-08

**Authors:** Sha Li, Laurien Oliver-Mighten, Lin Li, Sarah J. White, Kevin B. Paterson, Jingxin Wang, Kayleigh L. Warrington, Victoria A. McGowan

**Affiliations:** ^1^Academy of Psychology and Behavior, Tianjin Normal University, Tianjin, China; ^2^Department of Neuroscience, Psychology and Behaviour, University of Leicester, Leicester, United Kingdom

**Keywords:** aging, text spacing, eye movements, reading, word frequency

## Abstract

Large-scale changes in text spacing, such as removing the spaces between words, disrupt reading more for older (65+ years) than younger (18–30 years) adults. However, it is unknown whether older readers show greater sensitivity to simultaneous subtle changes in inter-letter and inter-word spacing encountered in everyday reading. To investigate this, we recorded young and older adults’ eye movements while reading sentences in which inter-letter and inter-word spacing was normal, condensed (10 and 20% smaller than normal), or expanded (10 or 20% larger than normal). Each sentence included either a high or low frequency target word, matched for length and contextual predictability. Condensing but not expanding text spacing disrupted reading more for the older adults. Moreover, word frequency effects (the reading time cost for low compared to high frequency words) were larger for the older adults, consistent with aging effects on lexical processing in previous research. However, this age difference in the word frequency effect did not vary across spacing conditions, suggesting spacing did not further disrupt older readers’ lexical processing. We conclude that visual rather than lexical processing is disrupted more for older readers when text spacing is condensed and discuss this finding in relation to common age-related visual deficits.

## Introduction

Considerable evidence shows that older adults (aged 65+ years) experience greater difficulty reading compared to young adults (aged 18–30 years), even when their visual and cognitive abilities appear normal (see Gordon et al., 2015). This is especially clear from studies of eye movements during reading, which consistently show that older adults read more slowly than young adults, by making more and longer fixations, and more backward eye movements, despite achieving normal comprehension (e.g., Kliegl et al., 2004; Rayner et al., 2006, 2009, 2011, 2013; Stine-Morrow et al., 2010; Paterson et al., 2013a,b,c; Jordan et al., 2014; McGowan et al., 2014, 2015; see also Whitford and Titone, 2016, 2017; Zang et al., 2016; Choi et al., 2017; Li et al., 2018; Wang et al., 2018a,b; Warrington et al., 2018). Some eye movement studies have also investigated adult age differences in the word frequency effect, which is the reading time cost for words that have a lower rather than higher frequency of written usage (e.g., Inhoff and Rayner, 1986; Rayner and Duffy, 1986; Rayner et al., 1996; White, 2008). These studies show a larger word frequency effect for older readers, due to older adults experiencing particular greater difficulty when identifying lower frequency words (Kliegl et al., 2004; Rayner et al., 2006; McGowan et al., 2015; Zang et al., 2016; Whitford and Titone, 2017; Wang et al., 2018a,b), consistent with the view that lexical processing is slower in older age (see Laubrock et al., 2006; Rayner et al., 2006).

The underlying causes of aging effects on eye movements during reading nevertheless are poorly understood although likely to be associated with declines in visual and cognitive abilities in later adulthood. Normative aging, in particular, is associated with both ocular and neural changes that can produce subtle reductions in visual functioning (see Owsley, 2011, 2016). These include reduced sensitivity to fine visual detail (e.g., edges, features; Owsley et al., 1983; Crassini et al., 1988; Elliott et al., 1995), which may lead older adults to rely more heavily on global cues during reading (Paterson et al., 2013b,c; Jordan et al., 2014), as well as increased effects of visual crowding, which is difficulty identifying visual objects in clutter (e.g., Bouma, 1970; Pelli and Tillman, 2008; Whitney and Levi, 2011; see Scialfa et al., 2013). Such deficits are likely to have important consequences for older adults’ reading behavior and, in particular, may lead older readers to be more sensitive to changes in the visual characteristics of text that affect the discriminability of letters and words.

An important strand of research has examined this issue by assessing adult age differences in the effects of removing the spaces between words. For many languages, including English, spaces are important for delineating the boundaries between words in text, and can both facilitate word recognition and help guide eye movements (Rayner et al., 1998). Removing (or filling) these spaces slows reading dramatically (e.g., Spragins et al., 1976; Malt and Seamon, 1978; Pollatsek and Rayner, 1982; Morris et al., 1990; Rayner et al., 1998, 2013; Perea and Acha, 2009; Paterson and Jordan, 2010; Sheridan et al., 2013; although see Epelboim et al., 1994, for claims for more modest effects). However, this slowing is even greater for older adults (Rayner et al., 2013; McGowan et al., 2014), suggesting that older readers benefit more from the use of inter-word spaces. Where studies have included a manipulation of word frequency (e.g., Rayner et al., 1998, 2013; McGowan et al., 2014), the findings show that removing spaces produces larger word frequency effects, by disproportionately increasing reading times for lower frequency words. However, studies that also included older participants show this influence of spacing on the word frequency effect is similar across adult age groups (Rayner et al., 2013; McGowan et al., 2014). The greater difficulty older readers experience with unspaced text therefore does not appear to be due to added disruption to lexical processing.

Unspaced text is not normally encountered (certainly when reading English). Therefore, other studies have investigated the influence of subtle variations in text spacing likely to occur more naturally in everyday reading, including due to font differences. This includes changes in the size of spaces between words and between the letters in words. Several studies present evidence that increases in inter-letter and inter-word spacing can benefit the recognition of both individually presented words and text reading by developing readers and children with developmental dyslexia, as well as typically developed young adults (Spinelli et al., 2002; Perea et al., 2011, 2012; Perea and Gomez, 2012a; Zorzi et al., 2012; see also Chung, 2002; Yu et al., 2007). Moreover, several eye movement studies show that increasing either only inter-letter spacing (Perea et al., 2016) or both inter-letter and inter-word spacing simultaneously produces shorter fixations on words (Perea and Gomez, 2012b; Slattery and Rayner, 2013). By comparison, simultaneously reducing both types of spacing is associated with longer fixations on words (Slattery and Rayner, 2013). These effects are usually explained in terms of the influence of spacing on visual crowding. In particular, it is argued that crowding is greater and impairs word recognition when spacing is decreased. By comparison, increased spacing is likely to decrease crowding compared to normal and so facilitate word recognition.

Importantly, increased spacing does not always produce faster sentence reading times, and did not do so in the eye movement studies reported by Perea and Gomez (2012b) and Slattery and Rayner (2013). This might be because sentences with increased inter-word or inter-letter spacing are physically longer and so need more fixations to be read. However, increased text spacing is also likely to impair parafoveal processing, which refers to the pre-processing of upcoming words outside of foveal vision to facilitate word recognition and guide eye movements (see Rayner, 1998, 2009; Schotter et al., 2012). As increased text spacing will serve to push these words further into extra-foveal vision, they are likely to be perceived at lower-acuity retinal regions and so processed less well (for discussion, see Slattery and Rayner, 2013). Any benefits of increased spacing for word recognition may therefore be offset by the impairment of normal parafoveal processing with the result that sentence reading is not benefited overall. Why increased text spacing does not also appear to decrease overall reading speeds in studies with developing readers or readers with developmental dyslexia is unclear but might be because these less-skilled readers engage in parafoveal processing to a much lesser degree than skilled adult readers.

Most previous studies also have not included older adults as participants and so are not informative about adult age differences in the effects of subtle changes in text spacing. Moreover, the only study to date that has done so looked only at inter-word spacing (McGowan et al., 2015). This showed that subtle increases in the spaces between words produced shorter fixations, whereas subtle decreases produced longer fixations. These effects were essentially the same for young and older adults, however, and neither increasing nor decreasing inter-word spacing influenced the word frequency effect for either age group. It therefore appears that increases in text spacing have no specific benefits for older readers. However, as this study did not also examine effects of changes in inter-letter spacing, further research is needed to more fully understand if older adults can benefit from simultaneous subtle increases in inter-letter and inter-word spacing similarly to young adults in previous eye movement studies (Perea and Gomez, 2012b; Slattery and Rayner, 2013).

Finally, we considered whether saccade-targeting adjusts to accommodate changes in the spatial extent of words when text spacing is varied. It is well-established that decisions about when and where to move the eyes are made independently (Rayner and McConkie, 1976). Moreover, readers move their eyes roughly from one word to the next (except when skipping words), rarely fixating the spaces between words, and targeting their saccades toward the center of upcoming words (Rayner, 1979; for a review, see Rayner, 1998, 2009). Additional evidence also suggests these aspects of eye movement control are preserved in older adults (Rayner et al., 2006; Paterson et al., 2013a). The present experiment allowed us to assess this matter further, by examining whether readers can adapt to changes in the length and location of words produced by variation in text spacing, and whether adaptability is observed for both young and older readers. In particular, it seemed likely that forward eye movements would be longer compared to normal when spacing is expanded, and shorter than normal when spacing is condensed. Moreover, text spacing may affect the likelihood of word-skipping. In particular, words might be skipped more often compared to normal when spacing is condensed, either because parafoveal processing is facilitated (Slattery and Rayner, 2013) or because condensed words provide poorer visual targets more likely to be overshot due to oculomotor error (Hautala et al., 2011). Conversely, longer words may be skipped less often compared to normal when spacing is expanded, either because parafoveal processing is reduced when spacing is increased, or because expanded words provide larger targets less likely to be overshot due to oculomotor error.

**Figure 1:**
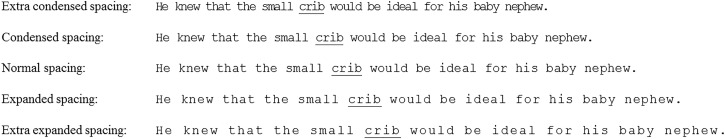
Example stimuli in each text spacing condition. The example includes a low frequency target word (“crib”) shown underlined. Target words were not underlined in the experiment.

We conducted an eye movement experiment to address the above issues. Young and older adults read sentences in which text spacing was normal, condensed so that it was 10 or 20% smaller than normal, or expanded so that it was 10 or 20% larger than normal (see Figure 1). The sentences also included one of a pair of target words that were high or low in lexical frequency and matched for length and predictability. We assessed age group differences in the effects of these manipulations of text spacing on sentence-level eye movement measures as well as effects of text spacing and lexical frequency on word-level eye movement measures for the target words.

We considered several key theoretical predictions. First, we expected to observe typical adult age differences in eye movements so that the older adults read sentences (and target words) more slowly, make more and longer fixations and more backward eye movements (regressions) compared to young adults. In addition, we expected to see typical effects of text spacing. In particular, in line with previous research, subtle increases in inter-letter and inter-word spacing might benefit eye movement behavior by reducing average fixation time and perhaps also producing faster sentences reading times, and these benefits may be greater for older adults. By comparison, condensed text might be detrimental to eye movement behavior, and so produce longer average fixations and perhaps also slower sentence reading times. Moreover, as older adults are affected more by visual crowding, and condensed text increases crowding effects, this disruption to normal reading might be greater for older adults. We also examined whether changes in text spacing affected saccade-targeting, by producing shorter forward eye movements but more word-skipping when spacing is condensed, but longer forward eye movements and less word-skipping when spacing is expanded, and whether these effects differ across adult age groups.

## Materials and Methods

### Participants

Participants were 20 young adults aged 18–32 years (*M* = 24 years) and 15 older adults aged 65–78 years (*M* = 71 years) from the University of Leicester community. All were native English speakers and the two age groups were closely matched for years of formal education [young adults, *M* = 16 years; older adults, *M* = 16; *t*(33) = 0.051, *p* > 0.1]. Finally, all participants reported reading for (at least) several hours per week.

Participants were screened for normal cognitive abilities using the Montreal Cognitive Assessment (applying a standard exclusion criterion of <26/30; Nasreddine et al., 2005), normal visual acuity (i.e., >20/40 in Snellen values) using an ETDRS (Early Treatment Diabetic Retinopathy Study) eye chart (Ferris and Bailey, 1996), and tested for contrast-sensitivity using a Pelli-Robson chart (Pelli et al., 1988). Compared to the young adults, the older adults had lower acuity [young adults, *M* = 20/17, range = 20/13–20/28; older adults, *M* = 20/29, range = 20/17–20/40; *t*(33) = 6.84, *p* < 0.001] and lower contrast-sensitivity [young adults, *M* = 1.97, range = 1.90–2.10; older adults, *M* = 1.83, range = 1.65–1.95; *t*(33) = 4.06, *p* < 0.001], as is typical for these age groups (Elliott et al., 1995). Tests of working memory and vocabulary capabilities were conducted using the Digit Span and Vocabulary subtests from WAIS-IV (Wechsler, 1997). The two age groups did not differ in digit span (young adults, *M* = 20; older adults, *M* = 21; *t* < 1.2; note values refer to test scores, not digit span), as is often reported for these age groups (e.g., Ryan et al., 2000; Bopp and Verhaeghen, 2005). However, vocabulary scores were higher for the older adults [young adults, *M* = 46; older adults, *M* = 51; *t*(33) = 2.6, *p* < 0.05, again, note values refer to test scores, not vocabulary size], consistent with a vocabulary advantage for older readers (Ben-David et al., 2015; Keuleers et al., 2015).

### Materials and Design

Stimuli were 60 sets of sentences, each containing one of a pair of interchangeable target words from an experiment by White et al. (2018). These words were either high or low in lexical frequency [high frequency words, *M* = 5.24, *SD* = 0.30; low frequency words, *M* = 3.36, *SD* = 0.32; *t*(59) = 30.50, *p* < 0.001] in Zipf-values from the SUBTLEX-UK databases (Van Heuven et al., 2014), and matched for letter length and predictability in the sentence context. The sentences averaged 61 characters including spaces (range = 53–67 characters), and the target word always appeared toward the middle of a sentence.

The sentences were displayed in 1 of 5 spacing conditions (see Figure 1): normal, extra-condensed (in which inter-letter and inter-word spaces were 20% smaller than normal), condensed (inter-letter and inter-word spaces 10% smaller than normal), expanded (inter-letter and inter-word spaces were 10% larger than normal), and extra-expanded (inter-letter and inter-word spaces 20% points larger than normal). The increases in inter-letter spacing were similar to those in the study by Perea and Gomez (2012b), and the increases in inter-word spacing were similar to those in the study by Perea et al. (2016) but more subtle than in the study by McGowan et al. (2015). Images were created using Microsoft PowerPoint.

The sentences were selected for display to each participant using a Latin square, so that each participant saw each sentence only once, in one spacing condition and word frequency condition, but sentences were shown equally often in each spacing and word frequency condition across each age group. Sentences were presented in random order in a single session, preceded by 10 practice sentences (two in each spacing condition). The experiment had a mixed design with the factors age group and text spacing in sentence-level analyses, and word-level analyses included the additional factor target word frequency.

### Apparatus and Procedure

A tower-mounted Eyelink 1000 eye-tracker (SR Research) recorded each participant’s right-eye gaze location every millisecond during binocular viewing. Sentence stimuli were presented as black text on a gray background (RGB: 240, 240, 240) in Courier New font on a 20-inch high-resolution monitor. At a viewing distance of 80 cm, three characters subtended approximately 1° in the normal spacing condition, and so were of normal size for reading (Rayner and Pollatsek, 1989).

Participants took part individually. At the start of the experiment, each participant was instructed to read normally and for comprehension. The eye-tracker was then calibrated to the participant’s eye movements using a 3-point horizontal calibration procedure and ensuring high spatial accuracy (<0.30° error). Calibration accuracy was checked before the start of each trial and the eye-tracker re-calibrated if spatial accuracy exceeded 0.30°. At the start of each trial, a fixation square was presented on the left side of the screen. Once the participant fixated this location, a sentence was presented with the first character replacing the square. Participants pressed a button on a response box once they finished reading each sentence. The sentence then disappeared and on 25% of trials was replaced by a two-alternative comprehension question requiring a “yes” or “no” response, to which the participant responded using one of two buttons. For each participant, the experiment lasted approximately 45 min.

## Results

Accuracy answering the comprehension questions that followed sentences was high for all participants (*M* = 93%) and did not differ across age groups or text spacing conditions (*p*s > 0.1). Both age groups could therefore comprehend the sentences well even when text spacing was condensed or expanded relative to normal.

Effects of text spacing on reading was assessed using standard sentence- and word-level measures (see Rayner, 1998, 2009). Sentence-level measures comprised sentence reading time (the time from the onset of a sentence display until the participant pressed a response key to indicate they had finished reading), average fixation duration (average length of fixations made during sentence reading), number of fixations (total number of fixations), number of regressions (number of backward eye movements), first-pass word-skipping (number of words not fixated prior to a fixation on a word to its right), and forward saccade length (the distance of progressive eye movements, reported here in degrees of visual angle, rather than characters, as changes in inter-letter spacing resulted in different character sizes across spacing conditions). Word-level measures assessed both the first-pass and later processing of the target words. First-pass measures were sensitive to effects occurring during the initial reading of a word prior to the eye leaving the word and moving to its right. We report the following first-pass measures: word skipping (probability of not fixating a word prior to an eye movement to its right), first-fixation duration (length of the first fixation on a word during first-pass reading), single-fixation duration (length of the fixation on a word receiving only one first-pass fixation), gaze duration (sum of all first-pass fixations on a word), and regressions out (probability of a first-pass regression from a word). We also report total reading time (sum of all fixations on a word) and regressions in (probability of a regression back to a word) as measures of later processing.

Following standard procedures, short (<80 ms) and long (>1200 ms) fixations were deleted (affecting 5.0% of fixations). Trials were also excluded if track loss or data-recording error occurred (affecting 0.2% of trials). The remaining data were analyzed using repeated measures analysis of variance (ANOVA) with factors age group and text spacing for sentence-level analyses, and including target word frequency for word-level analyses. Variance was computed across participants (*F*_1_) and stimuli (*F*_2_). Partial eta-squared ($\eta_{p}^{2}$) is reported as a measure of effect size. Pairwise comparisons were Bonferroni corrected.

### Sentence-Level Measures

Table 1 shows means for sentence-level measures and Table 2 reports ANOVA statistics.

**Table 1:** Mean sentence-level measures.

Measure	Age group	Extra-condensed	Condensed	Normal	Expanded	Extra-expanded
Sentence reading time (ms)	Young	2228 (194)	2148 (172)	2149 (160)	2245 (163)	2257 (168)
	Older	3400 (224)	3061 (198)	2884 (184)	2875 (189)	2977 (194)
Average fixation Duration (ms)	Young	212 (6)	207 (6)	201 (5)	197 (6)	196 (5)
	Older	269 (7)	254 (7)	245 (6)	240 (6)	236 (6)
Number of fixations	Young	9.3 (0.6)	9.2 (0.6)	9.4 (0.6)	9.8 (0.5)	10.0 (0.6)
	Older	12.0 (0.7)	11.4 (0.6)	11.2 (0.6)	11.4 (0.6)	11.9 (0.6)
Number of regressions	Young	1.8 (0.2)	1.8 (0.2)	1.7 (0.2)	1.8 (0.2)	1.8 (0.2)
	Older	2.9 (0.3)	2.5 (0.2)	2.3 (0.2)	2.3 (0.2)	2.4 (0.2)
First-pass word-skipping	Young	5.2 (0.3)	5.1 (0.3)	4.8 (0.3)	4.5 (0.3)	4.2 (0.3)
	Older	5.1 (0.3)	5.0 (0.3)	4.7 (0.3)	4.4 (0.3)	4.1 (0.3)
Forward saccade length (degrees)	Young	1.7 (0.1)	1.9 (0.1)	2.0 (0.1)	2.1 (0.1)	2.2 (0.1)
	Older	2.1 (0.1)	2.2 (0.1)	2.3 (0.1)	2.4 (0.1)	2.5 (0.1)

*Standard errors are shown in parentheses*.

**Table 2:** Statistical values for sentence-level measures.

	*F*_1_			*F*_2_		
	*df*	*F*	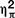	*df*	*F*	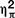
**Sentence reading time**						
Age group	1, 33	10.58**	0.24	1, 59	877.3***	0.94
Text spacing	4, 132	13.39***	0.29	4, 236	10.27***	0.15
Age group × Text spacing	4, 132	11.9***	0.27	4, 236	7.83***	0.12
**Average fixation duration**						
Age group	1, 33	29.48**	0.47	1, 59	10.91***	0.16
Text spacing	4, 132	83.39**	0.72	4, 236	65.71***	0.53
Age group × Text spacing	4, 132	9.13**	0.22	4, 236	1428.53***	0.96
**Number of fixations**						
Age group	1, 33	5.86*	0.15	1, 59	526.89***	0.90
Text spacing	4, 132	10.06**	0.23	4, 236	6.37***	0.10
Age group × Text spacing	4, 132	6.49**	0.16	4, 236	3.43**	0.06
**Number of regressions**						
Age group	1, 33	6.57*	0.17	1, 59	141.91***	0.71
Text spacing	4, 132	6.88**	0.17	4, 236	4.19**	0.07
Age group × Text spacing	4, 132	5.40**	0.14	4, 236	3.66**	0.06
**First-pass word-skipping**						
Age group	1, 33	0.09	0.003	1, 59	13.07**	0.18
Text spacing	4, 132	100.3***	0.75	4, 236	97.24***	0.62
Age group × Text spacing	4, 132	0.05	0.002	4, 236	0.07	0.001
**Forward saccade length**						
Age group	1, 33	7.26*	0.18	1, 59	1055.24***	0.95
Text spacing	4, 132	161.7***	0.83	4, 236	121.83***	0.67
Age group × Text spacing	4, 132	0.32	0.01	4, 236	0.47	0.01

*^∗^p < 0.05, ^∗∗^p < 0.01, ^∗∗∗^p < 0.001.*

A main effect of age group was obtained for all measures. Compared to the young adults, the older adults read more slowly, made more and longer fixations, more regressions, and longer forward saccades, but with no difference in first-pass word-skipping. These findings resonate with aging effects in previous research (e.g., Kliegl et al., 2004; Rayner et al., 2006, 2009, 2011, 2013; Stine-Morrow et al., 2010; Paterson et al., 2013a,b,c; Jordan et al., 2014; McGowan et al., 2014, 2015). The older adults therefore produced typical patterns of age-related reading difficulty. Main effects of text spacing were obtained for all sentence-level measures. Compared to the other spacing conditions, extra-condensed text produced the longest sentence reading times and average fixations and most regressions. Reading times were also longer than normal for sentences with extra-expanded spacing, most likely due to readers making more fixations when letters and words were further apart. Forward saccade length differed depending on the physical length of sentences, so that saccades were shorter than normal for condensed and extra-condensed sentences, but longer than normal for expanded and extra-expanded sentences. Finally, the number of words skipped during first-pass reading was higher than normal for condensed and extra-condensed sentences, and lower than normal for expanded and extra-expanded sentences. This can be explained in terms of either increased parafoveal processing or poorer saccade-targeting when text is condensed, and either decreased parafoveal processing and more accurate saccade-targeting when text is expanded. Crucially, these effects did not differ between young and older adults, suggesting that effects of text spacing on saccade-targeting was similar across adult age groups.

Importantly, main effects of text spacing in sentence reading times, average fixation durations, and numbers of fixations and regressions were qualified by interactions with age group. Pairwise comparisons showed that extra-condensed spacing slowed sentence reading times and lengthened average fixation durations compared to normal for both young and older adults, and that expanded text spacing slowed reading times compared to normal for the young adults but not the older adults. The older adults, but not young adults, also made more regressions when spaces were extra-condensed than in the other spacing conditions, suggesting older readers experienced greater difficulty when text was condensed. Finally, while older adults made more fixations for extra-condensed and extra-expanded sentences compared to the other spacing conditions, young adults made more fixations on expanded and extra-expanded sentences compared to the other spacing conditions.

To further explore these age differences in text spacing effects, we computed the size of the aging effect in each spacing condition. These values were entered into one-way repeated measures ANOVA with the variable text spacing (restricted to *F*_2_ analyses, as *F*_1_ analyses used a mixed design). This produced significant effects for all sentence-level measures [sentence reading times: *F*_2_(4,236) = 7.28, *p* < 0.001; average fixation durations: *F*_2_(4,236) = 10.91, *p* < 0.001; number of fixations: *F*_2_(4,236) = 3.43, *p* < 0.05; number of regressions: *F*_2_(4,236) = 3.19, *p* < 0.05]. Pairwise comparisons showed condensed and extra-condensed sentences produced the largest age difference in sentence reading times, and extra-condensed sentences produced the largest age difference in average fixation durations and numbers of fixations and regressions. This analysis therefore brings further clarity to the effects of text spacing across the two age groups by showing that disruption to normal reading was caused primarily by condensed text, and that this effect was larger for the older adults. The findings in addition show no evidence that increased text spacing benefited either age group.

### Target Word Analyses

Table 3 shows means for word-level measures and Table 4 reports ANOVA statistics.

**Table 3:** Mean word-level measures.

						Text spacing					
	
		Extra-condensed		Condensed		Normal		Expanded		Extra-expanded	
		
Measure	Age group	High	Low	High	Low	High	Low	High	Low	High	Low
Word-skipping (%)	Young	24 (3)	19 (4)	23 (4)	15 (3)	25 (4)	16 (3)	17 (3)	13 (3)	15 (3)	10 (3)
	Older	18 (3)	15 (4)	21 (4)	19 (4)	21 (5)	15 (3)	13 (4)	14 (3)	7 (3)	10 (3)
First-fixation duration (ms)	Young	220 (9)	247 (12)	222 (9)	237 (9)	215 (9)	235 (10)	207 (8)	233 (11)	214 (10)	237 (11)
	Older	276 (10)	305 (14)	261 (11)	283 (10)	235 (10)	266 (11)	236 (9)	265 (13)	236 (11)	260 (13)
Single-fixation duration (ms)	Young	222 (9)	248 (14)	223 (9)	238 (10)	216 (9)	236 (10)	207 (8)	234 (11)	216 (10)	240 (12)
	Older	281 (11)	316 (16)	261 (11)	277 (12)	236 (11)	268 (12)	238 (9)	271 (13)	238 (11)	266 (14)
Gaze duration (ms)	Young	230 (14)	276 (20)	234 (12)	255 (20)	224 (10)	256 (14)	216 (9)	254 (14)	224 (10)	260 (16)
	Older	317 (16)	377 (23)	275 (14)	345 (23)	254 (11)	291 (16)	253 (11)	301 (16)	255 (11)	301 (18)
Regression-out (%)	Young	6 (2)	13 (3)	8 (2)	8 (2)	5 (2)	6 (3)	9 (3)	4 (2)	3 (2)	4 (2)
	Older	17 (3)	17 (3)	9 (3)	10 (3)	9 (3)	11 (3)	9 (3)	8 (2)	6 (2)	10 (2)
Total reading Time (ms)	Young	250 (31)	323 (41)	253 (20)	300 (26)	237 (21)	285 (25)	239 (16)	297 (25)	244 (19)	283 (28)
	Older	440 (36)	583 (48)	351 (23)	476 (30)	315 (25)	411 (28)	329 (18)	392 (29)	326 (22)	433 (33)
Regressions-in (%)	Young	6 (2)	11 (4)	7 (2)	12 (3)	5 (3)	11 (4)	5 (2)	12 (2)	7 (2)	8 (3)
	Older	11 (3)	26 (4)	13 (3)	21 (4)	10 (3)	22 (5)	13 (3)	16 (3)	16 (3)	26 (3)

*Standard errors are shown in parentheses*.

**Table 4:** Statistical values for analyses of word-level measures.

Measure	*F*_1_			*F*_2_		
	*df*	*F*	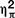	*df*	*F*	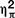
**Word-skipping**						
Age group	1, 33	0.50	0.02	1, 58	6.04*	0.09
Text spacing	4, 132	8.29***	0.20	4, 232	9.39***	0.14
Word frequency	1, 33	19.87***	0.38	1, 58	10.02**	0.15
Age group × Text spacing	4, 132	0.87	0.03	4, 232	1.09	0.02
Age group × Word frequency	1, 33	9.18**	0.22	1, 58	6.55*	0.10
Text spacing × Word frequency	4, 132	1.28	0.04	4, 232	0.99	0.02
Age group × Text spacing × Word frequency	4, 132	0.28	0.01	4, 232	0.21	0.00
**First-fixation duration**						
Age group	1, 33	7.75**	0.19	1, 58	115.92***	0.67
Text spacing	4, 132	13.23***	0.29	4, 232	11.7***	0.17
Word frequency	1, 33	103.92***	0.76	1, 58	109.24***	0.65
Age group × Text spacing	4, 132	5.04**	0.13	4, 232	3.71**	0.06
Age group × Word frequency	1, 33	0.86	0.03	1, 58	2.46	0.04
Text Spacing × Word frequency	4, 132	0.54	0.02	4, 232	0.35	0.01
Age group × Text spacing × Word frequency	4, 132	0.17	0.00	4, 232	0.22	0.00
**Single-fixation duration**						
Age group	1, 33	7.6**	0.19	1, 58	114.51***	0.7
Text spacing	4, 132	14.41***	0.30	4, 232	7.07***	0.12
Word frequency	1, 33	67.26***	0.67	1, 58	79.6***	0.61
Age group × Text spacing	4, 132	14.41**	0.30	4, 232	2.31†	0.04
Age group × Word frequency	1, 33	1.18	0.03	1, 58	1.47	0.03
Text spacing × Word frequency	4, 132	1.18	0.04	4, 232	0.18	0.00
Age group × Text spacing × Word frequency	4, 132	0.15	0.00	4, 232	0.18	0.00
**Gaze-duration**						
Age group	1, 33	9.62**	0.23	1, 58	134.02***	0.70
Text spacing	4, 132	16.02***	0.33	4, 232	13.13***	0.19
Word frequency	1, 33	54.47***	0.62	1, 58	99.4***	0.63
Age group × Text spacing	4, 132	7.43***	0.18	4, 232	5.09**	0.08
Age group × Word frequency	1, 33	2.25	0.06	1, 58	7.23**	0.11
Text spacing × Word frequency	4, 132	0.60	0.02	4, 232	0.26	0.00
Age group × Text spacing × Word frequency	4, 132	1.01	0.03	4, 232	0.96	0.02
**Regressions-out**						
Age group	1, 33	57.32***	0.64	1, 58	9.16**	0.14
Text spacing	4, 132	8.00***	0.20	4, 232	5.00**	0.08
Word frequency	1, 33	0.63	0.02	1, 58	1.48	0.03
Age group × Text spacing	4, 132	1.58	0.05	4, 232	0.89	0.02
Age group × Word frequency	1, 33	0.09	0.00	1, 58	0.58	0.01
Text spacing × Word frequency	4, 132	1.42	0.04	4, 232	1.09	0.02
Age group × Text spacing × Word frequency	4, 132	0.82	0.02	4, 232	0.19	0.00
**Total reading time**						
Age group	1, 33	15.86***	0.33	1, 58	216.43***	0.79
Text spacing	4, 132	16.06***	0.33	4, 232	15.35***	0.21
Word frequency	1, 33	95.99***	0.74	1, 58	62.63***	0.52
Age group × Text spacing	4, 132	8.60***	0.21	4, 232	8.82***	0.13
Age group × Word frequency	1, 33	10.67**	0.24	1, 58	10.99**	0.16
Text spacing × Word frequency	4, 132	1.94	0.06	4, 232	0.79	0.01
Age group × Text spacing × Word frequency	4, 132	1.28	0.04	4, 232	0.72	0.01
**Regressions-in**						
Age group	1, 33	12.55**	0.28	1, 58	44.83***	0.44
Text spacing	4, 132	0.88	0.03	4, 232	0.92	0.02
Word frequency	1, 33	43.98***	0.57	1, 58	27.37***	0.32
Age group × Text spacing	4, 132	1.46	0.04	4, 232	1.16	0.02
Age group × Word frequency	1, 33	4.34*	0.12	1, 58	4.84*	0.08
Text spacing × Word frequency	4, 132	0.68	0.02	4, 232	0.33	0.01
Age group × Text spacing × Word frequency	4, 132	1.03	0.03	4, 232	1.32	0.02

*^†^p< 0.1, ^∗^p < 0.05, ^∗∗^p < 0.01, ^∗∗∗^p < 0.001.*

Main effects of age group in all measures except word-skipping showed that fixation times were longer and regression rates higher for the older than younger adults, resonant with findings from previous research (Kliegl et al., 2004; Rayner et al., 2006, 2009, 2011, 2013; Stine-Morrow et al., 2010; Paterson et al., 2013a,b,c; Jordan et al., 2014; McGowan et al., 2014, 2015). Main effects of word frequency in all measures are also in line with well-established findings showing that higher frequency words are skipped less often and receive shorter reading times and fewer regressions than lower frequency words (e.g., Inhoff and Rayner, 1986; Rayner and Duffy, 1986; Rayner et al., 1996; White, 2008). An interaction between age group and word frequency in total reading times was due to a larger word frequency effect for the older adults. This pattern is in line with previous research indicating that older readers have disproportionately greater difficulty processing lower frequency words, although the effect in the present experiment emerged later in the eye movement record than in previous studies (Kliegl et al., 2004; Rayner et al., 2006, 2013; McGowan et al., 2015; Whitford and Titone, 2017). This age difference in the word frequency effect may have been driven primarily by the re-inspection of words. In particular, as older readers are more likely to re-inspect words, they may have experienced a “double whammy” effect when first encountering and then re-reading lower frequency words, thereby inflating their reading times for these words. However, whether the relatively late appearance of this age difference in the word frequency effect was due to the manipulation of word spacing is unclear. Finally, an interaction between age group and word frequency in word-skipping was due to a larger increase in skipping for high compared to low frequency words for the young adults than older adults. This may be because the young adults, but not the older adults, could use parafoveal processing to facilitate the recognition of higher frequency words.

Main effects of text spacing were observed in all eye-movement measures except regressions-in. These effects were qualified by interactions between age group and text spacing in first-fixation duration, single-fixation duration (*F*_1_ analysis only), gaze duration and total reading time. The pattern of effects was very similar across these measures, and so we will refer to this effect collectively as an effect on reading times. For the young adults, condensing or expanding text had little influence on reading times compared to normal. For the older adults, expanding text also had little influence on reading times compared to normal. However, condensing text (especially in the extra-condensed condition) produced longer than normal reading times for the older adults. As before, aging effects were explored further by conducting supplementary analyses that compared age differences in the size of the spacing effect for each reading time measure (restricted to *F*_2_ analyses). This produced significant effects for all measures [first-fixation duration: *F*_2_(4,236) = 4.31, *p* < 0.01; single-fixation duration: *F*_2_(4,236) = 3.69, *p* < 0.01; gaze duration: *F*_2_(4,236) = 6.42, *p* < 0.001; total reading times: *F*_2_(4,236) = 8.79, *p* < 0.001]. Pairwise comparisons indicated that these were due to larger age differences for extra-condensed text compared to normal (significant in gaze durations and total reading times, marginal in first-fixation durations). This analysis therefore brought clarity to our findings by revealing that age differences primarily were due to older adults having particular difficulty when reading extra-condensed text.

Returning to the main ANOVA results, we observed a main effect of text spacing in regressions-out due to more regressions for extra-condensed spacing compared to the other display conditions, which did not differ significantly from each other. We also observed an effects of text spacing on word-skipping that did not interact with age group. Pairwise comparisons showed word-skipping was lowest for expanded and extra-expanded spacing and equally higher for normal, condensed and extra-condensed spacing. The effect for expanded text was similar to that observed in skipping effects in the sentence-level analyses, and so provided further evidence that text spacing affected saccade-targeting. As the effect was essentially the same for young and older adults, the finding also provides further evidence that this effect of spacing on saccade-targeting is similar across adult age groups. Finally, the main ANOVA analyses produced no two-way interactions between text spacing and word frequency, and no three-way interactions. Consequently, effects of text spacing did not appear to influence the word frequency effect for either age group.

## Discussion

The present study provides valuable evidence concerning effects of aging and text spacing on eye movements during reading. First, the findings showed clear patterns of age-related reading difficulty, resonant with findings from previous research (e.g., Kliegl et al., 2004; Rayner et al., 2006, 2009, 2011, 2013; Stine-Morrow et al., 2010; Paterson et al., 2013a,b,c; Jordan et al., 2014; McGowan et al., 2014, 2015; Whitford and Titone, 2016, 2017; Warrington et al., 2018). As in this previous research, the older adults read more slowly and made more and longer fixations and more regressions than young adults. The older adults therefore exhibited typical patterns of age-related reading difficulty. They also made longer forward eye movements than young adults, consistent with findings from previous research showing that older adults compensate for slower reading by leaping ahead in the text more often (see Rayner et al., 2006). However, in contrast with previous research, this did not cause older adults to skip words more frequently.

The findings also show effects of text spacing on eye movement behavior. In particular, condensing or expanding the spacing between letters and words increased sentence reading times and disrupted normal eye movement behavior. Crucially, these effects differed for the young and older adults. For the young adults, both condensing and expanding text spacing resulted in longer sentence reading times (due to more than normal fixations and longer than normal average fixation durations when text was condensed, and more than normal numbers of fixations when text was expanded). However, for the older adults, disruption to normal reading primarily was observed for condensed text, which produced longer than normal sentence reading times as well as more than normal numbers of fixations and longer than normal average fixations durations. Supplementary analyses clarified these findings by showing that age differences in text spacing effects were primarily due to condensed text causing greater disruption for older readers. Analyses that examined the processing of specific target words in sentences (for which we also manipulated lexical frequency) confirmed this key finding, by showing increases in word reading times were larger for the older adults when spacing was condensed. Therefore, while there was little indication that increased spacing benefited reading for either age group, condensed spacing clearly caused greater disruption for the older readers. Such effects may be due to increased crowding when text is condensed. We discuss these findings further in relation to previous research below.

The analysis of the high and low frequency target words in the sentences showed standard word frequency effects for both age groups, so that both had lower word-skipping rates and longer fixation times for low compared to high frequency words. Previous research has shown that older readers tend to produce larger word frequency effects due to disproportionately longer reading times for low compared to high frequency words (Kliegl et al., 2004; Rayner et al., 2006, 2013; McGowan et al., 2015; Whitford and Titone, 2017; Wang et al., 2018a,b). An age difference in the word frequency effect was also observed in the present research, although this emerged late in the eye movement record (in total reading times rather than gaze durations on target words). We suggested it may reflect a “double whammy” effect whereby older readers had difficulty both when first encountering a low frequency word and during its subsequently re-reading, thereby producing a larger word frequency effect overall. Despite the difference in the time course of this effect compared to in previous research, the finding is nevertheless consistent with the view that older readers lexically process words more slowly (e.g., Laubrock et al., 2006; Rayner et al., 2006). Crucially, text spacing did not influence the size of the word frequency effect for either age group, suggesting that subtle variation in spacing did not impact on the lexical processing of words. This is in line with other evidence showing that variation in text spacing has little influence on the word frequency effect (Perea and Gomez, 2012b; Slattery and Rayner, 2013; McGowan et al., 2015). However, it contrasts with findings showing that unspaced text produces a larger than normal word frequency effect for both young and older adult readers (Rayner et al., 2013; McGowan et al., 2014). The overall indication, therefore, is that lexical identification can proceed as normal for both young and older readers so long as word boundaries can be delineated.

Finally, effects of text spacing on forward saccade length and word-skipping showed that decisions about where to move the eyes were influenced by the physical extent of words and the sizes of spaces between words. The findings for saccade length showed that saccades were shorter when text was condensed, but longer when text was expanded. As sentences with different spacing were inter-mixed in the experiment, this finding is consistent with other evidence that saccade length adjusts to changes in average word length (Cutter et al., 2017, 2018), and that saccadic control is driven by the physical characteristics of words. Word-skipping also differed as a function of text spacing, so that skipping rates were higher than normal for condensed text, and lower than normal for expanded text. Such effects can be explained in terms of parafoveal processing or the likelihood of a saccade overshooting a word target. In particular, increased skipping of condensed words might reflect enhanced parafoveal processing (Slattery and Rayner, 2013) or because physically shorter words provide poorer saccade targets, consistent with evidence that word skipping is largely driven by a word’s spatial extent rather than its number of letters (Hautala et al., 2011). Conversely, less skipping of expanded words might be because parafoveal processing is poorer when text is expanded, or because physically longer words provide better saccade targets. Crucially, as these effects did not differ across age groups, the present findings also provide further evidence that motor control of the eyes during reading is preserved in older age (Rayner et al., 2006; Paterson et al., 2013a).

Our findings make a useful contribution to our understanding of text spacing effects on eye movements during reading. First, they add to evidence of a trade-off in the benefits and disadvantages of increased text spacing in natural reading. As in the present experiment, several studies have shown that increased spacing can shorten fixations on words but also increase the overall number of fixations and sometimes also the time taken to read sentences (Perea and Gomez, 2012b; Slattery and Rayner, 2013; McGowan et al., 2015; Perea et al., 2016). Such effects are attributed to increased spacing facilitating the recognition of fixated words while simultaneously impairing parafoveal processing by causing upcoming text to be perceived at lower acuity retinal regions (for discussion, see Slattery and Rayner, 2013). Consequently, while there may be specific benefits of increasing text spacing for the recognition of individual words, these may be offset by other disadvantages for skilled reading. The present research shows a very similar pattern of effects for young and older adults and so it seems unlikely that increasing text spacing will provide an effective method of ameliorating age-related reading difficulty.

The present findings also suggest that while subtle increases in text spacing can affect the visual processing of text, it may do so without affecting the lexical identification of words. That is to say, there was no evidence that variation in text spacing differentially affected the processing of high and low frequency words in the present experiment. This finding is consistent with other evidence that subtle variation in text spacing has little influence on the word frequency effect in reading (Perea and Gomez, 2012b; Slattery and Rayner, 2013; McGowan et al., 2015). However, as already noted, disruption to the word frequency effect is well-established when spaces between words are missing (Rayner et al., 2013; McGowan et al., 2014). It therefore seems likely that lexical identification can proceed as normal so long as visual cues to word boundaries are available.

Finally, the present study adds to our understanding of age differences in text spacing effects by showing eye movements are disrupted more for older readers when inter-letter and inter-word spacing is (simultaneously) condensed relative to normal. This is consistent with the view that older readers may be especially sensitive to changes in the visual characteristics of text that affect the discriminability of letters and words (see also Rayner et al., 2013; McGowan et al., 2014). Note, however, that this contrasts with the McGowan et al. (2015) findings that condensed text produces few adult age differences in eye movement behavior. However, this may be because McGowan et al. manipulated only inter-word spacing, whereas we manipulated both inter-word and inter-letter spacing, and because changes in inter-letter spacing were primarily responsible for the effects we observed. If correct, this suggests that condensing letter spacing caused older readers particular difficulty, possibly due to increased effects of visual crowding. Further work is needed to clarify this influence of text spacing. But, if correct, such findings may have implications for the selection of fonts to support older adult reading, as spacing between letters in fonts is highly variable and typically larger in non-proportional fonts in which each letter occupies the same horizontal space compared to proportional fonts where letters vary in the horizontal space they occupy. Consequently, an important objective of future research may be to establish whether fonts with greater inter-letter spacing provide better support for older readers compared to fonts in which inter-letter spacing is condensed.

## Ethics Statement

This study was carried out in accordance with the recom-mendations of University of Leicester’s Code of Practice for Research Ethics with written informed consent from all subjects. All subjects gave written informed consent in accordance with the Declaration of Helsinki. The protocol was approved by the University Ethics Sub-Committee of Psychology at the University of Leicester.

## Author Contributions

SL is lead author. JW and VM are joint corresponding authors. SL and LO-M collected the data. SL and VM analyzed the data. SL, KP, JW, and VM wrote the manuscript. All authors contributed to the concept and design of the experiments.

## Conflict of Interest Statement

The authors declare that the research was conducted in the absence of any commercial or financial relationships that could be construed as a potential conflict of interest.

## References

[B1] Ben-David B. M. Erel H. Goy H. Schneider B. A. (2015). “Older is always better”: age-related differences in vocabulary scores across 16 years. *Psychol. Aging* 30 856–862. 10.1037/pag0000051 26652725

[B2] Bopp K. L. Verhaeghen P. (2005). Aging and verbal memory span: a meta-analysis. *J. Gerontol. B Psychol. Sci. Soc. Sci.* 60 223–233. 10.1093/geronb/60.5.P223 16131616

[B3] Bouma H. (1970). Interaction effects in parafoveal letter recognition. *Nature* 226 177–178. 10.1038/226177a0 5437004

[B4] Choi W. Lowder M. W. Ferreira F. Swaab T. Y. Henderson J. M. (2017). Effects of word predictability and preview lexicality on eye movements during reading: a comparison between young and older adults. *Psychol. Aging* 32 232–242. 10.1037/pag0000160 28333501

[B5] Chung S. T. L. (2002). The effect of letter spacing on reading speed in central and peripheral vision. *Invest. Ophthalmol. Vis. Sci.* 43 1270–1276. 11923275

[B6] Crassini B. Brown B. Bowman K. (1988). Age-related-changes in contrast sensitivity in central and peripheral retina. *Perception* 17 315–332. 10.1068/p170315 3067210

[B7] Cutter M. G. Drieghe D. Liversedge S. P. (2017). Reading sentences of uniform word length: evidence for the adaptation of the preferred saccade length during reading. *J. Exp. Psychol.* 43 1895–1911. 10.1037/xhp0000416 28406688

[B8] Cutter M. G. Drieghe D. Liversedge S. P. (2018). Reading sentences of uniform word length - II: very rapid adaptation of the preferred saccade length. *Psychon. Bull. Rev.* 25 1435–1440. 10.3758/s13423-018-1473-2 29696593 PMC6096651

[B9] Elliott D. B. Yang K. C. Whitaker D. (1995). Visual acuity changes throughout adulthood in normal, healthy eyes: seeing beyond 6/6. *Optom. Vis. Sci.* 72 186–191. 10.1097/00006324-199503000-00006 7609941

[B10] Epelboim J. Booth J. R. Steinman R. M. (1994). Reading unspaced text: implications for theories of reading eye movements. *Vis. Res.* 34 1735–1766. 10.1016/0042-6989(94)90130-9 7941379

[B11] Ferris F. L. Bailey I. L. (1996). Standardizing the measurement of visual acuity for clinical research studies. *Ophthalmology* 103 181–182. 10.1016/S0161-6420(96)30742-2 8628551

[B12] Gordon P. C. Lowder M. W. Hoedemaker R. S. (2015). “Reading in normally aging adults,” in *Cognitive-Linguistic Processes and Aging*, ed. Wright H. H. (Amsterdam: John Benjamins Publishing), 165–191.

[B13] Hautala J. Hyönä J. Aro M. (2011). Dissociating spatial and letter-based word length effects observed in readers’ eye movement patterns. *Vis. Res.* 51 1719–1727. 10.1016/j.visres.2011.05.015 21664920

[B14] Inhoff A. W. Rayner K. (1986). Parafoveal word processing during eye fixations in reading: effects of word frequency. *Percept. Psychophys.* 40 431–439. 10.3758/BF03208203 3808910

[B15] Jordan T. R. McGowan V. A. Paterson K. B. (2014). Reading with filtered fixations: age differences in the effectiveness of low-level properties of text within central vision. *Psychol. Aging* 29 229–235. 10.1037/a0035948 24955991

[B16] Keuleers E. Stevens M. Mandera P. Brysbaert M. (2015). Word knowledge in the crowd: measuring vocabulary size and word prevalence in a massive online experiment. *Q. J. Exp. Psychol.* 68 1665–1692. 10.1080/17470218.2015.1022560 25715025

[B17] Kliegl R. Grabner E. Rolfs M. Engbert R. (2004). Length, frequency, and predictability effects of words on eye movements in reading. *Eur. J. Cogn. Psychol.* 16 262–284. 10.1080/09541440340000213

[B18] Laubrock J. Kliegl R. Engbert R. (2006). SWIFT explorations of age differences in eye movements during reading. *Neurosci. Biobehav. Rev.* 30 872–884. 10.1016/j.neubiorev.2006.06.013 16904181

[B19] Li S. Li L. Wang J. McGowan V. A. Paterson K. B. (2018). Effects of word length on eye guidance differ for young and older Chinese readers. *Psychol. Aging* 33 685–692. 10.1037/pag0000258 29902059 PMC6001944

[B20] Malt B. C. Seamon J. G. (1978). Peripheral and cognitive components of eye guidance in filled-space reading. *Percept. Psychophys.* 23 399–402. 10.3758/BF03204142 683824

[B21] McGowan V. A. White S. J. Jordan T. R. Paterson K. B. (2014). Aging and the use of interword spaces during reading: evidence from eye movements. *Psychon. Bull. Rev.* 21 740–747. 10.3758/s13423-013-0527-8 24101571

[B22] McGowan V. A. White S. J. Paterson K. B. (2015). The effects of interword spacing on the eye movements of young and older readers. *J. Cogn. Psychol.* 27 609–621. 10.1080/20445911.2014.988157

[B23] Morris R. K. Rayner K. Pollatsek A. (1990). Eye guidance in reading: the role of parafoveal letter and space information. *J. Exp. Psychol. Hum. Percept. Perform.* 16 268–281. 10.1037/0096-1523.16.2.268 2142198

[B24] Nasreddine Z. S. Phillips N. A. Bédirian V. Charbonneau S. Whitehead V. Collin I. (2005). The Montreal Cognitive Assessment, MoCA: a brief screening tool for mild cognitive impairment. *J. Am. Geriatr. Soc.* 53 695–699. 10.1111/j.1532-5415.2005.53221.x 15817019

[B25] Owsley C. (2011). Aging and vision. *Vis. Res.* 51 1610–1622. 10.1016/j.visres.2010.10.020 20974168 PMC3049199

[B26] Owsley C. (2016). Vision and aging. *Annu. Rev. Vis. Sci.* 14 255–271. 10.1146/annurev-vision-111815-114550 28532355

[B27] Owsley C. Sekuler R. Siemsen D. (1983). Contrast sensitivity throughout adulthood. *Vis. Res.* 23 689–699. 10.1016/0042-6989(83)90210-9 6613011

[B28] Paterson K. B. Jordan T. R. (2010). Effects of increased letter spacing on word identification and eye guidance during reading. *Mem. Cogn.* 38 502–512. 10.3758/MC.38.4.502 20516230

[B29] Paterson K. B. McGowan V. A. Jordan T. R. (2013a). Aging and the control of binocular fixations during reading. *Psychol. Aging* 28 789–795. 10.1037/a0033328 23978007

[B30] Paterson K. B. McGowan V. A. Jordan T. R. (2013b). Effects of adult aging on reading filtered text: evidence from eye movements. *PeerJ* 1:e63 10.7717/peerj.63 PMC3642708 23646282

[B31] Paterson K. B. McGowan V. A. Jordan T. R. (2013c). Filtered text reveals adult age differences in reading: evidence from eye movements. *Psychol. Aging* 28 352–364. 10.1037/a0030350 23066801

[B32] Pelli D. G. Robson J. G. Wilkins A. J. (1988). The design of a new letter chart for measuring contrast sensitivity. *Clin. Vis. Sci.* 2 187–199. 11428528

[B33] Pelli D. G. Tillman K. A. (2008). The uncrowded window of object recognition. *Nat. Neurosci.* 11 1129–1135. 10.1038/nn.2187 18828191 PMC2772078

[B34] Perea M. Acha J. (2009). Space information is important for reading. *Vis. Res.* 49 1994–2000. 10.1016/j.visres.2009.05.009 19463847

[B35] Perea M. Giner L. Marcet A. Gomez P. (2016). Does extra interletter spacing help text reading in skilled adult readers? *Span. J. Psychol.* 19:E26. 10.1017/sjp.2016.28 27210581

[B36] Perea M. Gomez P. (2012a). Increasing interletter spacing facilitates encoding of words. *Psychon. Bull. Rev.* 19 332–338. 10.3758/s13423-011-0214-6 22351586

[B37] Perea M. Gomez P. (2012b). Subtle increases in interletter spacing facilitate the encoding of words during normal reading. *PLoS One* 7:e47568. 10.1371/journal.pone.0047568 23082178 PMC3474730

[B38] Perea M. Moret-Tatay C. Gomez P. (2011). The effects of interletter spacing in visual-word recognition. *Acta Psychol.* 137 345–351. 10.1016/j.actpsy.2011.04.003 21545978

[B39] Perea M. Panadero V. Moret-Tatay C. Gomez P. (2012). The effects of inter-letter spacing in visual-word recognition: evidence with young normal readers and developmental dyslexics. *Learn. Instr.* 22 420–430. 10.1016/j.learninstruc.2012.04.001

[B40] Pollatsek A. Rayner K. (1982). Eye movement control in reading: the role of word boundaries. *J. Exp. Psychol. Hum. Percept. Perform.* 8 817–833. 10.1037/0096-1523.8.6.817

[B41] Rayner K. (1979). Eye guidance in reading: fixation locations within words. *Perception* 8 21–30. 10.1068/p080021 432075

[B42] Rayner K. (1998). Eye movements in reading and information processing: 20 years of research. *Psychol. Bull.* 124 372–422. 10.1037/0033-2909.124.3.372 9849112

[B43] Rayner K. (2009). The thirty-fifth sir frederick bartlett lecture: eye movements and attention in reading, scene perception, and visual search. *Q. J. Exp. Psychol.* 62 1457–1506. 10.1080/17470210902816461 19449261

[B44] Rayner K. Castelhano M. S. Yang J. (2009). Eye movements and the perceptual span in older and younger readers. *Psychol. Aging* 24 755–760. 10.1037/a0014300 19739933

[B45] Rayner K. Duffy S. A. (1986). Lexical complexity and fixation times in reading: effects of word frequency, verb complexity, and lexical ambiguity. *Mem. Cogn.* 14 191–201. 10.3758/BF03197692 3736392

[B46] Rayner K. Fischer D. L. Pollatsek A. (1998). Unspaced text interferes with both word identification and eye movement control. *Vis. Res.* 38 1129–1144. 10.1016/S0042-6989(97)00274-5 9666972

[B47] Rayner K. McConkie G. W. (1976). What guides a reader’s eye movements? *Vis. Res.* 16 829–837. 10.1016/0042-6989(76)90143-7 960610

[B48] Rayner K. Pollatsek A. (1989). *The Psychology of Reading.* Englewood Cliffs, NJ: Prentice Hall.

[B49] Rayner K. Reichle E. Stroud M. Williams C. Pollatsek A. (2006). The effect of word frequency, word predictability, and font difficulty on the eye movements of young and older readers. *Psychol. Aging* 21 448–465. 10.1037/0882-7974.21.3.448 16953709

[B50] Rayner K. Sereno S. C. Raney G. E. (1996). Eye movement control in reading: a comparison of two types of models. *J. Exp. Psychol. Hum. Percept. Perform.* 22 1188–1200. 10.1037/0096-1523.22.5.1188 8865619

[B51] Rayner K. Yang J. Castelhano M. S. Liversedge S. P. (2011). Eye movements of older and younger readers when reading disappearing text. *Psychol. Aging* 26 214–223. 10.1037/a0021279 21142374

[B52] Rayner K. Yang J. Schuett S. Slattery T. J. (2013). Eye movements of older and younger readers when reading unspaced text. *Exp. Psychol.* 60 354–361. 10.1027/1618-3169/a000207 23681016

[B53] Ryan J. J. Sattler J. M. Lopez S. J. (2000). Age effects on wechsler adult intelligence scale-III subtests. *Arch. Clin. Neuropsychol.* 15 311–317. 10.1093/arclin/15.4.311 14590227

[B54] Schotter E. R. Angele B. Rayner K. (2012). Parafoveal processing in reading. *Attent. Percept. Psychophys.* 74 5–35. 10.3758/s13414-011-0219-2 22042596

[B55] Scialfa C. T. Cordazzo S. Bubric K. Lyon J. (2013). Aging and visual crowding. *J. Gerontol. Ser. B Psychol. Sci. Soc. Sci.* 68 522–528. 10.3758/s13414-011-0219-2 23009956

[B56] Sheridan H. Rayner K. Reingold E. M. (2013). Unsegmented text delays word identification: evidence from a survival analysis of fixation durations. *Vis. Cogn.* 21 38–60. 10.1080/13506285.2013.767296

[B57] Slattery T. J. Rayner K. (2013). Effects of intraword and interword spacing on eye movements during reading: exploring the optimal use of space in a line of text. *Attent. Percept. Psychophys.* 75 1275–1292. 10.3758/s13414-013-0463-8 23709061

[B58] Spinelli D. De Luca M. Judica A. Zoccolotti P. (2002). Crowding effects on word identification in developmental dyslexia. *Cortex* 38 179–200. 10.1016/S0010-9452(08)70649-X 12056688

[B59] Spragins A. B. Lefton L. A. Fisher D. F. (1976). Eye movements while reading and searching spatially transformed text: a developmental examination. *Mem. Cogn.* 4 36–42. 10.3758/BF03213252 21286956

[B60] Stine-Morrow E. A. L. Shake M. C. Miles J. R. Lee K. Gao X. McConkie G. (2010). Pay now or pay later: aging and the role of boundary salience in self-regulation of conceptual integration in sentence processing. *Psychol. Aging* 25 168–176. 10.1037/a0018127 20230137 PMC2841323

[B61] Van Heuven W. J. B. Mandera P. Keuleers E. Brysbaert M. (2014). Subtlex-UK: a new and improved word frequency database for British english. *Q. J. Exp. Psychol.* 67 1176–1190. 10.1080/17470218.2013.850521 24417251

[B62] Wang J. Li L. Li S. Xie F. Chang M. Paterson K. B. (2018a). Adult age differences in eye movements during reading: the evidence from Chinese. *J. Gerontol. Ser. B Psychol. Sci. Soc. Sci.* 73 584–593. 10.1093/geronb/gbw036 27032427 PMC6019021

[B63] Wang J. Li L. Li S. Xie F. Liversedge S. P. Paterson K. B. (2018b). Effects of aging and text stimulus quality on the word frequency effect during Chinese reading. *Psychol. Aging* 33 693–712. 10.1037/pag0000259 29781642

[B64] Warrington K. L. McGowan V. A. Paterson K. B. White S. J. (2018). Effects of aging, word frequency and text stimulus quality on reading across the adult lifespan: evidence from eye movements. *J. Exp. Psychol. Learn. Mem. Cogn.* 44 1714–1729. 10.1037/xlm0000543 29672115 PMC6233613

[B65] Wechsler D. (1997). *WAIS-III Administration and Scoring Manual.* San Antonio, TX: The Psychological Corporation.

[B66] White S. J. (2008). Eye movement control during reading: effects of word frequency and orthographic familiarity. *J. Exp. Psychol. Hum. Percept. Perform.* 34 205–223. 10.1037/0096-1523.34.1.205 18248149

[B67] White S. J. Drieghe D. Liversedge S. P. Staub A. (2018). The word frequency effect during sentence reading: a linear or nonlinear effect of log frequency? *Q. J. Exp. Psychol.* 71 46–55. 10.1080/17470218.2016.1240813 27760490

[B68] Whitford V. Titone D. (2016). Eye movements and the perceptual span during first- and second-language sentence reading in bilingual older adults. *Psychol. Aging* 31 58–70. 10.1037/a0039971 26866589

[B69] Whitford V. Titone D. (2017). The effects of word frequency and word predictability during first- and second-language paragraph reading in bilingual older and younger adults. *Psychol. Aging* 32 158–177. 10.1037/pag0000151 28287786

[B70] Whitney D. Levi D. M. (2011). Visual crowding: a fundamental limit on conscious perception and object recognition. *Trends Cogn. Sci.* 15 160–168. 10.1016/j.tics.2011.02.005 21420894 PMC3070834

[B71] Yu D. Y. Cheung S. H. Legge G. E. Chung S. T. L. (2007). Effect of letter spacing on visual span and reading speed. *J. Vis.* 7 2.1–210. 10.1167/7.2.2 18217817 PMC2729067

[B72] Zang C. Zhang M. Bai X. Yan G. Paterson K. B. Liversedge S. P. (2016). Effects of word frequency and visual complexity on eye movements of young and older Chinese readers. *Q. J. Exp. Psychol.* 69 1409–1425. 10.1080/17470218.2015.1083594 26366620

[B73] Zorzi M. Barbiero C. Facoetti A. Lonciari I. Carrozzi M. Montico M. (2012). Wider letter spacing helps dyslexic readers. *Proc. Natl Acad. Sci. U.S.A.* 109 11455–11459. 10.1073/pnas.1205566109 22665803 PMC3396504

